# Mitigating of Thin-Film Composite PTMSP Membrane Aging by Introduction of Porous Rigid and Soft Branched Polymeric Additives

**DOI:** 10.3390/membranes13010021

**Published:** 2022-12-23

**Authors:** Danila S. Bakhtin, Alexander O. Malakhov, Alexey V. Volkov, Leonid A. Kulikov, Inna V. Petrova, Ilya L. Borisov, Stepan D. Bazhenov

**Affiliations:** 1 A.V. Topchiev Institute of Petrochemical Synthesis, Russian Academy of Sciences, Leninsky Prospect 29, 119991 Moscow, Russia; 2 Biological and Environmental Science, and Engineering Division (BESE), Advanced Membranes and Porous Materials Center (AMPM), King Abdullah University of Science and Technology, Thuwal 23955, Saudi Arabia; 3 Chemistry Department, Moscow State University, Leninskie Gory 1/3, 119991 Moscow, Russia

**Keywords:** carbon dioxide, thin-film composite membrane, gas permeability, aging, PTMSP, porous aromatic framework, cross-linked PEI

## Abstract

This work was focused on the mitigation of physical aging in thin-film composite (TFC) membranes (selective layer ~1 μm) based on polymer intrinsic microporosity (PTMSP) by the introduction of both soft, branched polyethyleneimine (PEI), and rigid, porous aromatic framework PAF-11, polymer additives. Self-standing mixed-matrix membranes of thicknesses in the range of 20–30 μm were also prepared with the same polymer and fillers. Based on 450 days of monitoring, it was observed that the neat PTMSP composite membrane underwent a severe decline of its gas transport properties, and the resultant CO_2_ permeance was 14% (5.2 m^3^ (STP)/(m^2^·h·bar)) from the initial value measured for the freshly cast sample (75 m^3^ (STP)/(m^2^·h·bar)). The introduction of branched polyethyleneimine followed by its cross-linking allowed to us to improve the TFC performance maintaining CO_2_ permeance at the level of 30% comparing with day zero. However, the best results were achieved by the combination of porous, rigid and soft, branched polymeric additives that enabled us to preserve the transport characteristics of TFC membrane as 43% (47 m^3^ (STP)/(m^2^·h·bar) after 450 days) from its initial values (110 m^3^ (STP)/(m^2^·h·bar)). Experimental data were fitted using the Kohlrausch–Williams–Watts function, and the limiting (equilibrium) values of the CO_2_ and N_2_ permeances of the TFC membranes were estimated. The limit value of CO_2_ permeance for neat PTMSP TFC membrane was found to be 5.2 m^3^ (STP)/(m^2^·h·bar), while the value of 34 m^3^(STP)/(m^2^·h·bar) or 12,600 GPU was achieved for TFC membrane containing 4 wt% cross-linked PEI, and 30 wt% PAF-11. Based on the N_2_ adsorption isotherms data, it was calculated that the reduction of the free volume was 1.5–3 times higher in neat PTMSP compared to the modified one. Bearing in mind the pronounced mitigation of physical aging by the introduction of both types of fillers, the developed high-performance membranes have great potential as support for the coating of an ultrathin, selective layer for gas separation.

## 1. Introduction

Carbon dioxide capture and storage (CCS) is considered as the most promising technology for reducing carbon dioxide emissions from large stationary thermal power plants. At the same time, membrane technologies are considered as one of the promising approaches for capturing CO_2_, instead of the traditional alkanolamine absorption purification of gases from acidic impurities [1,2]. In this regard, many research groups all over the world are actively working on the development of new materials and high-performance membranes [3], applying of commercial membranes available on the market [4], and conducting pilot tests of tailor-made high-performance composite membranes [5,6,7,8] for CO_2_ capture from different gas streams with specific focus on flue gas treatment. It should be noted that the membrane performance parameters used for gas and liquid separation can be tuned by introduction of different kinds of additives [9,10].

Industrial gas-separation membranes are mostly based on glassy polymers due to their good balance between selectivity and permeability as well as their ability to withstand processing conditions [11]. At the same time, glassy polymers are nonequilibrium materials. They are prone to physical aging, during which their transport and other properties relax to an equilibrium state [12]. The relaxation of the nonequilibrium free volume is accompanied by a decrease in the gas permeability of membranes with time. Merkel et al. [5] have shown that, under practical conditions for post-combustion carbon capture, higher permeance dominates the economics of the separation process. High-free-volume glassy polymers are highly permeable to CO_2_ which makes them attractive materials for CO_2_ capture membrane development. However, the effect of “aging” is especially pronounced for membranes based on glassy polymers with a high free volume, for example, poly(1-trimethylsilyl-1-propyne) (PTMSP), poly-4-methylpentine-1 (PMP), polybenzodioxane (PIM-1), Teflon AF, Hyflon AD, and polynorbornens [11,12,13,14,15,16,17,18,19,20,21,22,23,24,25,26,27,28,29,30,31,32,33,34,35,36,37,38,39,40,41,42,43,44].

In this regard, several approaches such as the addition of non-porous and porous materials [13,14,16,17,20,22,23,24,25,32,33,39], post-modification of the prepared membranes via polymer cross-linking [21,28,31], a combination of cross-linking and addition of nanoparticles [19,35,36,38,39,40], and changes in the polymer backbone design have been proven to be controlling in the aging in high-free-volume glassy polymers. It should be emphasized that the vast majority of studies deal with dense membranes with a thickness of 30 µm or more. At the same time, high-free-volume glassy polymers can be applied for the fabrication of composite membranes with a thin selective layer. These can be either selective layers or gutter layers of composite membranes. It should be noted that in the development of TFC membranes for post-combustion CO_2_ capture, little attention is focused on the optimization of the membrane supports that satisfy the conditions of this technology.

With this in mind, Bazhenov et al. [31] developed a PTMSP-based TFC membrane suitable as a highly permeable and solvent-resistant support. Crosslinking of PTMSP was performed using polyethyleneimine and poly (ethyleneglycol) diglycidyl ether as crosslinking agents. Optimal concentrations of polyethyleneimine in PTMSP and poly (ethyleneglycol) diglycidyl ether in methanol were selected in order to diminish the undesirable effect on the final membrane gas transport characteristics. Later the negative effect of filler incorporation on the example of a three-layer TFC membrane, with PIM-1 + MOF composite as a selective layer was described by Liu et al. [37]. It is important to note that the PDMS + MOF gutter layer provided less gas transport resistance in comparison with pristine PDMS gutter layers.

It is known that glassy polymers such as polyphenylene oxide, polysulfone, polyimide, perfluoropolymers, and polyacetylenes in the form of thin films, with a thickness of up to a few μm, undergo accelerated physical aging [15]. In addition, Yavari et al. [34] investigated the effect of accelerated physical aging of the two-layer thin-film composite (TFC) membranes consisting of perfluoropolymers (including Teflons AF1600 and Hyflons AD) at various film thicknesses (50–400 nm) on polyethersulfone porous support. The permeances of CH_4_, N_2_, H_2_, and CO_2_ at 35 °C for over 1000 h were studied. It was demonstrated that the decrease of gas permeances with time was more significant for larger penetrants and membranes with thinner selective layers. For example, CH4 permeance decreased by 54% and 27% after aging for about 1400 h in TFC membranes comprising 50 nm and 370 nm Teflon AF1600, respectively.

Therefore, it is important to study the stabilization effect of different fillers on the transport properties of selective layers in TFC membranes. However, there is a very limited number of works devoted to the search for ways to reduce the aging effect for TFC membranes based on high-free-volume glassy polymers [35,36,37,38,39,40,41], namely, PIM-1 [37,41] and PTMSP [35,36,38,39,40].

In our previous work [35], the prevention of physical aging of TFC membranes based on PTMSP by the introduction of porous aromatic framework particles (PAF-11) was studied. A number of the TFC membranes with a varied thickness of the selective layer of 1.7–6.8 μm were fabricated, and their gas transport properties were monitored for up to 650 days at ambient temperature. Among all studied TFC membranes, the TFC/6.8/PAF sample provided better stability of gas permeance by retaining 0.19, 0.24, and 0.30 from its original values for N_2_, O_2_, and CO_2_, respectively. The best gas permeances for the stabilized aged TFC membrane were 0.8 (N_2_), 1.4 (O_2_), and 5.1 m^3^(STP)/(m^2^·h·bar), (CO_2_), and the ideal selectivity of 1.8 and 6.9 for the pair of O_2_/N_2_ and CO_2_/N_2_, respectively. We have also previously shown [36,38,40] that the TFC membranes with the selective layer based on crosslinked PTMSP/PEI containing 10 wt% of porous aromatic frameworks (PAF-11) nanoparticles demonstrated an improved gas permeance stability compared to the TFC membranes without PAF-11 filler. Therefore, this work is devoted to further improvement of TFC membrane stability within the framework of the developed approach. With this in mind, composite membranes with a thin (1 μm) selective PTMSP/PEI layer containing 20 and 30 wt% of PAF-11 nanoparticles were obtained, and their gas transport properties for CO_2_ and N_2_ were monitored during 450 days at ambient temperature. Using the Kohlrausch–Williams–Watts function the limiting (equilibrium) values of the CO_2_ and N_2_ permeances of the TFC membranes were estimated.

## 2. Materials and Methods

### 2.1. Membrane Preparation

PTMSP (catalyst TaCl_5_/TIBA) was synthesized in the Laboratory of Synthesis of Selectively Permeable Polymers (TIPS RAS) according to the method described in [26]. Synthesis of porous aromatic frameworks PAF-11 was performed via Suzuki cross-coupling reaction between tetrakis-(p-bromophenyl)methane and 4,4′-biphenyldiboronic acid according the method described elsewhere [45].

As was shown in our previous work for dense films [31], as the concentration of PEI in PTMSP increases, crosslinking of the PTMSP/PEI mixture was shown to reduce the resultant CO_2_ permeance of membranes. All the crosslinked membranes were insoluble in chloroform. Hence, the crosslinking system containing 4 wt% PEI in PTMSP was selected as the optimal option due to the minimal turndown of membrane gas permeance. Bearing this in mind, the casting PTMSP solutions containing 4 wt% of polyethyleneimine (PEI) (Mw = 25,000 g/mol, Mn = 10,000 g/mol (Sigma-Aldrich, St. Louis, MO, USA)) relative to the weight of PTMSP were prepared by mixing of 0.5 wt% solutions of the individual polymers in chloroform. PAF-11 in the required amount was added to the PTMSP casting solution directly.

The thin layer of PTMSP was cast on the porous microfiltration support MFFK-1 with the mean pore size 0.15 μm (Vladipor, Russia) from 0.5 wt% polymeric solution in chloroform by a kiss-coating technique described elsewhere [46,47]. Composite membranes of 10 × 30 cm were fabricated. The freshly prepared membranes were immersed in 4 wt% solution of the poly(ethylene glycol) diglycidyl ether (PEGDGE, M ~ 500 g/mol, Sigma-Aldrich, USA) in methanol to cross-link the PEI amino groups in accordance with the protocol described earlier [31]. The duration of the post-modification process was not less than 72 h at ambient conditions. Then, for the removal of the residual PEGDGE and tangential stress relaxation, the membranes were immersed in n-butanol for 24 h, and then in 25 wt% ethanol aqueous solutions for the next 24 h. Before any measurements, the samples were dried in ambient conditions for at least 24 h. The prepared membranes PTMSP/PEI, PTMSP/PEI/20% PAF-11, and PTMSP/PEI/30% PAF-11 are designated for brevity as M0, M20, and M30, respectively.

**Figure 1:**
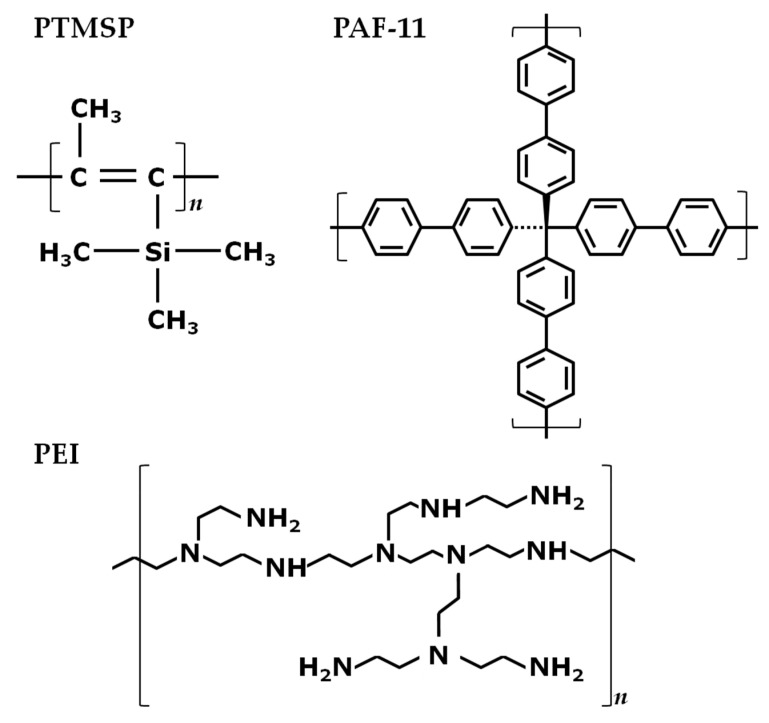
Chemical structure of polymers poly(1-trimethylsilyl-1-propyne), branched polyethyleneimine, and porous aromatic framework PAF-11.

For additional tests by helium pycnometer and nitrogen adsorption, samples of dense membranes (20 μm) were also prepared from the same casting solutions that were used for the composite membrane fabrication. Samples of dense membranes based on PTMSP were obtained by slow evaporation of the solvent (chloroform) from a cellophane substrate covered with a Petri dish [26]. The films prepared were dried for at least 7 days to constant weight at room temperature and atmospheric pressure. After complete drying, the samples were post-treated identically as in the case of TFC membranes: 72 h 4 wt% PEGDGE in methanol, 24 h in n-butanol, 24 h in 25 wt% ethanol aqueous solution, 24 h drying at ambient conditions. The chemical structures of PTMSP, PEI, and PAF-11 are shown in Figure 1.

### 2.2. Characterization

#### 2.2.1. Gas Permeability

Individual carbon dioxide and nitrogen permeances of TFC membranes were measured by the variable volume/constant pressure method at room temperature (22 ± 2 °C) with an error of ±10%. The membrane area was 12.6 cm^2^ [35]. The feed pressure was 3 bar and the permeate pressure was atmospheric. The permeance of TFC membranes was determined as

(1) $$Q\equiv J/\Delta p=\frac{1}{A\;\Delta p}\cdot \frac{dV}{dt}\cdot \frac{T_{\mathrm{SPT}}p^{perm}}{Tp_{\mathrm{SPT}}}$$

where *J* is the permeate flux per the membrane area *A*, $\Delta p=p^{feed}-p^{perm}$ is the transmembrane pressure ($p^{feed}$ and $p^{perm}$ are feed and permeate pressure, respectively), *T* is the absolute temperature, $T_{\mathrm{SPT}}$ and $p_{\mathrm{SPT}}$ are the standard temperature and pressure, and dV/dt is the volumetric flow rate on the downstream side of the membrane. The aging of the membranes was monitored by momentary measurements of gas permeance over 15 months. The ideal CO_2_/N_2_ selectivity was calculated as the ratio of CO_2_ and N_2_ permeances.

#### 2.2.2. Nitrogen Adsorption

The polymeric films (thickness 20–30 μm) were characterized by low-temperature N_2_ adsorption using a Gemini VII 2390 (Micromeritics) surface-area analyzer. Prior to the analysis, samples were degassed at 0.01 mbar overnight. The adsorption parameters were calculated from the isotherm data using the BET equation [48]:

(2) $$\frac{p/p_{0}}{v(1-p/p_{0})}=\frac{1}{v_{m}C_{\mathrm{BET}}}+\frac{C_{\mathrm{BET}}-1}{v_{m}C_{\mathrm{BET}}}p/p_{0}$$

where *v* is the adsorbed amount (cm^3^ (STP)/g), $v_{m}$ is the monolayer capacity (cm^3^ (STP)/g), $C_{\mathrm{BET}}$ is a constant characterizing the magnitude of the attractive adsorbate–adsorbent interaction, and $p/p_{0}$ is the relative vapor pressure. The BET adsorption parameters were determined in the relative pressure range of 0.001–0.2, in which experimental data depend linearly on the relative pressure according to Equation (2).

The specific surface area (m^2^/g) is proportional to the monolayer capacity and is defined as

(3) $$S_{\mathrm{BET}}=v_{m}\;a_{m}N_{A}\cdot 10^{-20}/22414=4.353\;v_{m}$$

where *N*_A_ is the Avogadro constant and *a_m_* is the molecular cross-sectional area (16.2 Å^2^ for nitrogen). 

The Henry constant (in units mol/g) is given by

(4) $$K_{H}=v_{m}C_{\mathrm{BET}}/22414$$

The unrelaxed free volume or ‘micropore volume’ per 1 g of an adsorbent was evaluated as

(5) $$W=\frac{v_{m}}{22414}\frac{M_{N_{2}}}{\rho_{N_{2}}}$$

where $M_{N_{2}}$ is the nitrogen molar mass (g/mol) and $\rho_{N_{2}}$ is *N*_2_ adsorbate density which was taken the value 0.8 g/cm^3^ (adsorbate density assumed to be comparable to the density of the liquid). Then, the free-volume fraction of a sample was evaluated as

(6) $$f=W\rho_{sk}/\left(1+W\rho_{sk}\right)$$

where $\rho_{sk}$ is the skeletal volume (g/cm^3^).

#### 2.2.3. Carbon Dioxide Exposure

CO_2_ exposure of the dense PTMSP-based films was carried out in a dead-end filtration cell. The samples were placed into the upper compartment of the cell and pressurized using CO_2_. The pressure in the cell was increased from 1 to 3 bar within 1 min and held constant for 20 days at room temperature.

#### 2.2.4. Helium Pycnometry

The helium pycnometer Micro-Ultrapyc 1200e (Quantachrome Instruments, Boynton Beach, FL, USA) was used to obtain the skeletal density of PTMSP-based films at T = 20.0 ± 0.5 °C.

#### 2.2.5. Scanning Electron Microscopy (SEM)

All obtained TFC membranes were analyzed by SEM. A high-resolution scanning electron microscope, Hitachi Tabletop Microscope TM3030Plus, was used. Beforehand, the samples were immersed in i-propanol to fill up the pore structure, then fractured in liquid nitrogen and sputtered under a vacuum with a thin (5 nm) layer of gold. For each composite membrane, 3 images of the cross-section were taken from different regions of the sample and 5 thickness measurements in each of the images were made. The deviation of the layer thickness did not exceed 0.1 μm.

#### 2.2.6. Solvent Exposure

To check short-time stability of the developed composite membranes in solvents the specimens of all membranes investigated were exposed in chloroform. Prepared as for cross-section SEM micrographs, the samples of all membranes were placed in chloroform for 60 s. After extraction, the samples were dried at room temperature and then examined by SEM.

#### 2.2.7. Processing of Gas-Permeability Experimental Data

The gas-permeability experimental data were fitted with the relaxation function φ(t), which describes the kinetics of the approach of the system to the equilibrium state. This function is defined as

(7) $$Q(t)=Q_{eq}+\left[Q(0)-Q_{eq}\right]\varphi (t)$$

where *Q*(*t*) is the permeance (generally, it is any glass material parameter, e.g., volume, enthalpy) at the time *t*, *Q*(0) is the initial permeance, and $Q_{eq}$ is the hypothetical limited value of *Q* at infinite time, i.e., the (metastable) equilibrium permeance. The relaxation function changes from 1 at start time to 0 when equilibrium is reached

The stretched exponential function was used as the relaxation function:

(8) $$\varphi (t)=\exp\left[-{\left(t/\tau \right)}^{\beta }\right]$$

with the parameters *τ* and *β* (0<β≤1). This analytical fitting function, commonly known as the Kohlrausch–Williams–Watts function, is often used to describe the relaxation properties (specific volume, enthalpy, etc.) of polymer glasses [12,49]. The stretching exponent *β* characterizes the width of the relaxation spectrum and *τ* is the time when the relaxation function takes l/*e* of its initial value [50].

Equation (7) may be written via the relative quantities

(9) $$q(t)=q_{eq}+(1-q_{eq})\exp\left[-{\left(t/\tau \right)}^{\beta }\right]$$

where

$$q(t)=Q(t)/Q(0),\;q_{eq}=Q_{eq}/Q(0)$$

Equation (9) includes three parameters: *τ*, *β*, and ‘equilibrium’ permeance $q_{eq}$. Obviously, the $q_{eq}$ value is not available from the experiment (the fully relaxed membranes are beyond the range of experimentally convenient times), so its estimation is useful for understanding how the state of polymer glass is far from the equilibrium. Note that such a three-parameter equation with the stretched exponential was first applied by Cowie and Ferguson to describe enthalpy relaxation in glassy polymers [50,51].

## 3. Results

### 3.1. Structure and Morphology

**Figure 2:**
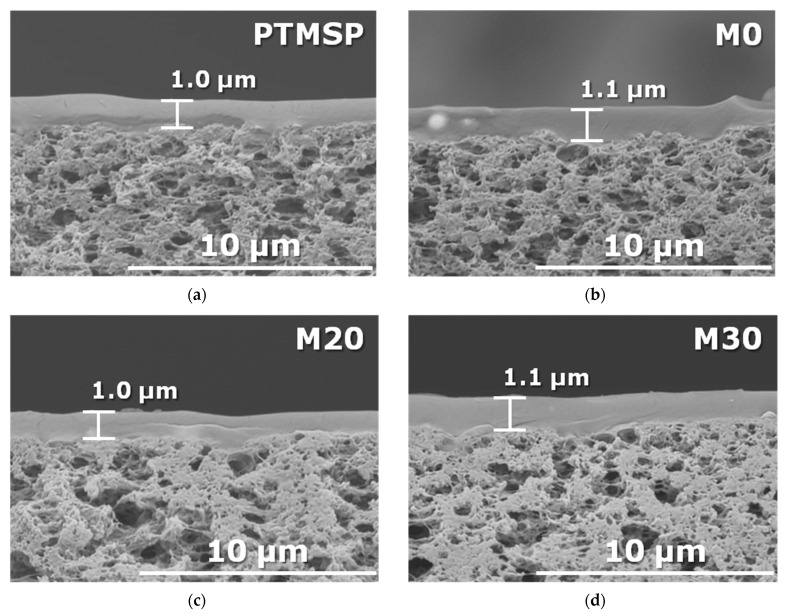

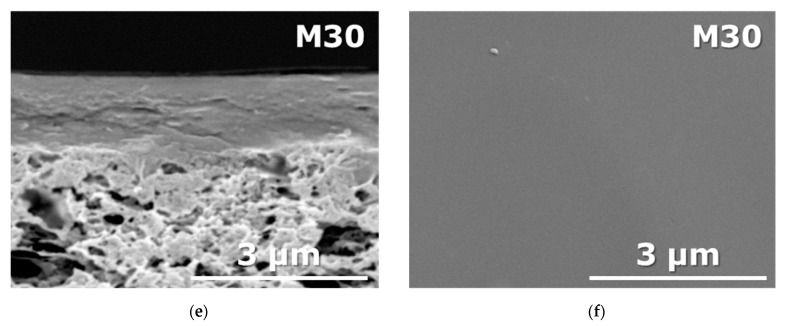
SEM images of the cross-section of TFC membranes: (**a**) neat PTMSP, (**b**) M0, (**c**) M20, (**d**,**e**) M30; and surface of M30 (**f**).

The cross-sectional SEM images of membranes are shown in Figure 2. It can be seen that the PAF-11 particles are mostly well distributed within the PTMSP polymer matrix due to the hydrophobic nature of porous particles and polymer material, and no noticeable aggregation of these particles was observed for all TFC membranes obtained. The average thickness of the selective layer for the samples studied was 1.0 ± 0.1 μm.

**Figure 3:**
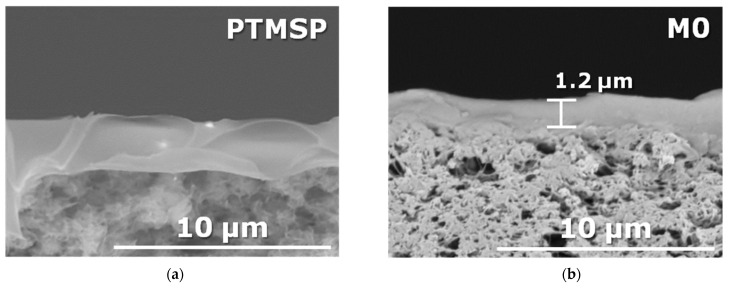

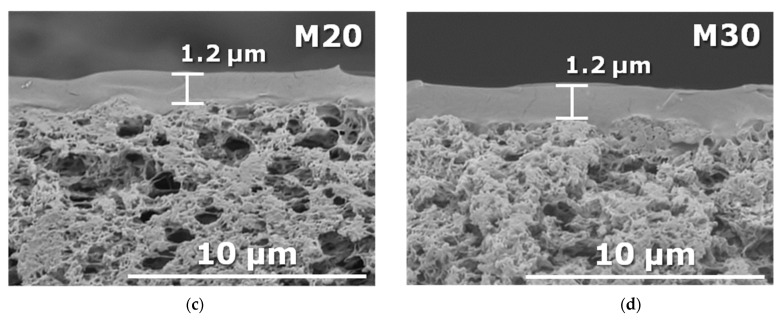
SEM images of the cross-section of TFC membranes after 60 s exposition in chloroform: (**a**) a neat PTMSP, (**b**) M0, (**c**) M20, and (**d**) M30 cross-section.

One possible application of the developed membranes is non-porous high flux support for the thin-film selective layer coating. With this in mind, samples of the TFC membranes (50 × 10 mm) were immersed for 60 s in chloroform. Multilayer composite membranes can be fabricated by sequential application of several layers one after another. In this case, the intermediate layer must be resistant to the composition of the casting solution of the subsequently applied layer [42]. In the case of the kiss-coating method, the contact time of the intermediate layer (gutter layer) material and the casting solution of the subsequent layer is short (a few seconds). Therefore, 60 s is enough time to check the short-time stability of the developed composite membranes in solvents. As an example, Figure 3a shows the SEM images of the composite membranes after 60 s exposure to chloroform. It can be seen that no selective layer deterioration or its detachment from porous MFFK support was observed for samples M0, M20, or M30 containing cross-linked PEI in a PTMSP matrix. However, a noticeable swelling of the selective layer was observed in the case of neat PTMSP composite membrane. A good stability was also observed for the samples M0, M20, and M30 after their 60 s exposure to hexane and toluene. Thus, the top layer of the samples M0, M20, and M30 can be used as a gutter layer for the further deposition of the CO_2_-selective materials from the organic solvents.

### 3.2. Aging of PTMSP-Based TFC Membranes

**Figure 4:**
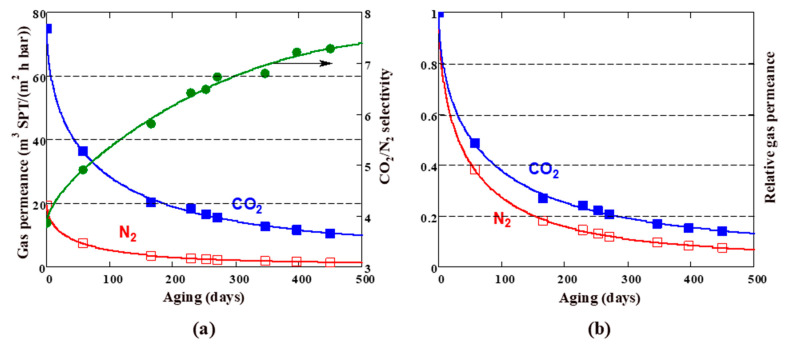
Absolute (**a**) and relative (**b**) CO_2_ and N_2_ permeances for TFC membrane with PTMSP selective layer vs. aging time. The CO_2_/N_2_ selectivity is also shown. Points, experiment; lines, approximation using Equation (9).

TFC membranes were monitored by N_2_ and CO_2_ gas permeance periodically within 15 months. The membranes were stored in a sealed bag between the measurements. The time dependence of the gas permeance through the TFC membrane with neat PTMSP selective layer is shown in Figure 4a. Predictably, both CO_2_ and N_2_ permeances decreased over time. The relative permeance (the ratio of the permeance at that time to the initial permeance) after 450 days was 14% and 8% for CO_2_ and N_2_, respectively. The CO_2_/N_2_ selectivity increased over the same time from 3 to 8.

The CO_2_ permeability loss for the unmodified PTMSP selective layer (86% in 450 days) is significant, but typical for high-free-volume glassy polymers [26,35]. For comparison, the loss of CO_2_ permeability for a thick PTMSP film (thickness ~100 μm) was 74% in 365 days [23]. Taking into account that the thickness of the selective layer in the considered TFC membranes is about 1 µm, as well as the known fact of accelerated aging of thin films [52,53,54], the obtained data on the aging of PTMSP seem to be quite reasonable. According to the generally accepted views [54,55], the decrease in gas permeability through PTMSP and other glassy polymers occurs due to the loss of excess free volume over time.

**Figure 5:**
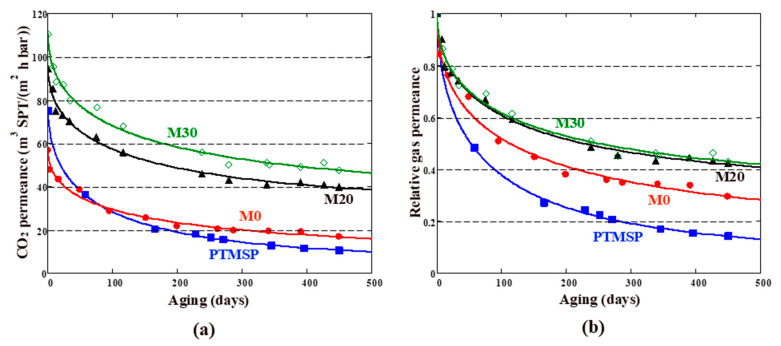
Absolute (**a**) and relative (**b**) CO_2_ permeances for TFC membranes with PTMSP and PTMSP/PEI/PAF-11 selective layer vs. aging time. Membranes M0, M20, and M30 contain 0, 20 and 30% porous filler PAF-11, respectively. Points, experiment; lines, approximation using Equation (9).

The long-term behavior of TFC membranes, doped with cross-linked PEI and PAF-11, is shown in Figure 5b. The CO_2_ permeance decreased with aging time. As can be seen, TFC membranes M0, M20, and M30 exhibited slower aging and correspondingly higher permeance compared to a TFC membrane with a neat PTMSP selective layer. Relative CO_2_ permeances of the membranes decreased in the series: PTMSP < M0 < M20 < M30. Note that the M0 membrane does not contain PAF-11 and yet showed twice the relative permeance over 450 days compared to the PTMSP membrane. This suggests that the insertion of crosslinked branched PEI into the PTMSP matrix leads to a certain stabilization of permeation behavior. The addition of porous filler PAF-11 into the cross-linked PTMSP/PEI matrix further slowed the relaxation of TFC membranes: the relative CO_2_ permeances were 42 and 43% over 450-day aging for membranes M20 and M30, respectively. Thus, blending PTMSP with PAF-11 allowed for increasing of the relative CO_2_ permeance by 28–29% compared to the neat PTMSP composite membrane. The reason for the permeability increase is probably associated with an increase in free-volume fraction.

The absolute values of permeances for TFC membranes M20 and M30 after 450 days were 39 and 47 m^3^(STP)/(m^2^·h·bar) (Figure 5a). These values exceed the previously obtained results for similar membranes containing 10 wt% PAF-11 (14.8 m^3^(STP)/(m^2^·h·bar)) [40]. The absolute values of gas permeances exceeded by several times the permeances for previously obtained hybrid composite membranes: PTMSP doped with 10 wt% PAF-11 on porous PAN support [35].

The parameters of Equation (9) were obtained by approximating the raw data and are listed in Table 1. We found that the stretching exponent *β* ranged from 0.49 to 0.56. These are reasonable values considering that the *β* parameter in the Kohlrausch–Williams–Watts function is usually less than 0.6 for the glassy amorphous polymers [49]. The relaxation time *τ* for both permeating gases (N_2_ and CO_2_) decreased in the series PTMSP < M0 < M20 < M30, i.e., it changed in the same order as the permeance of the corresponding membranes. Indeed, the shorter the relaxation time, the higher the aging rate, the faster the gas permeance should decrease. The limiting (t→∞) relative gas permeances for N_2_ and CO_2_ are given in the two last columns in Table 1. It can be seen that M20 and M30 membranes showed the same limiting relative CO_2_ permeability (*q_eq_* = 31%), which is noticeably (4.4 times) higher than the corresponding value for the unmodified PTMSP membrane. The absolute values (*Q_eq_*) of equilibrium CO_2_ permeance were 29 and 34 m^3^(STP)/(m^2^·h·bar) for the M20 and M30 membranes, respectively. The “equilibrium” CO_2_/N_2_ selectivity (Table 1) was 6 and 7 for the M20 and M30 membranes, respectively.

Table 2 shows comparative aging data for TFC membranes based on high permeability glassy polymers. It can be seen that PTMSP-based membranes with a high PAF-11 content were the least susceptible to physical aging. A smaller relative decrease in gas permeability was achieved only for a composite membrane based on PIM-1 by Bhavsar et al. during a 90-day aging study [41], while in the present work, a decrease in membrane gas transport performance was observed for 450 days. At a time interval of 90 days, the M30 membrane demonstrated a decrease in gas permeability of 30% from the initial value, compared to decreases of 19% for composite material of hypercrosslinked polystyrene and PIM-1. Since the selective layer of the membrane based on PIM-1 [41] is twice as thick as that of M30 and, therefore, is less prone to aging, it can be argued that the introduction of 4% cross-linked PEI and 30% PAF-11 into the matrix of highly permeable glassy polymers is one of the most effective anti-aging methods for TFC membranes.

### 3.3. N_2_ Adsorption

Nitrogen adsorption at 77 K is the widely used method of characterization of micro- and mesoporous solids. Theoretically, PAF-11 has an expanded diamond-like structure, which should lead to the formation of a mesoporous material with a long-range order. However, the distortion and interpenetration of the aromatic building units leads to a dramatic decrease in the framework porosity [56,57], so in reality PAF-11 is an amorphous material with a predominantly microporous network structure.

**Figure 6:**
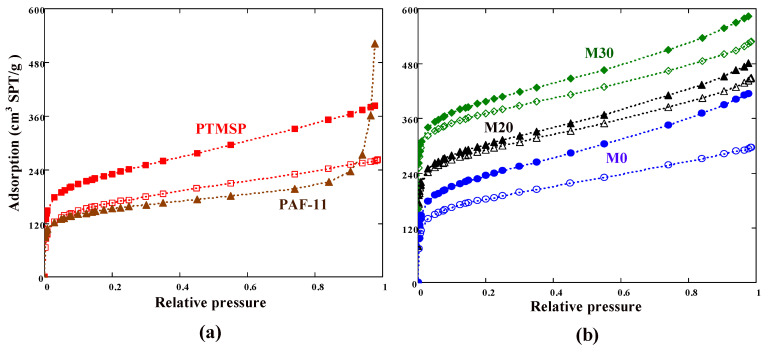
(**a**) Adsorption isotherms of nitrogen on PAF-11 and PTMSP samples and (**b**) adsorption isotherms of nitrogen on M0 (PTMSP/PEI), M20 (PTMSP/PEI/20% PAF-11), and M30 (PTMSP/PEI/30% PAF-11) samples. The isotherms for M20 and M30 were shifted by 80 and 160 cm^3^ STP/g, respectively. Closed and open symbols denote PTMSP-based samples before and after CO_2_ exposure, respectively.

Nitrogen isotherms for polymeric films containing PAF-11 additive were obtained both for as-cast polymer films and for films that were exposed to CO_2_ for a long time (20 days) at a pressure of 3 bar. Adsorption isotherms (Figure 6) exhibited a sharp uptake at low relative pressures; the relative pressure corresponding to the monolayer capacity was for all polymeric films; for the porous filler PAF-11 this value was even lower and was about 0.02. The N_2_ adsorption for PTMSP-based films exceeded the amount adsorbed for porous filler PAF-11 up to the relative pressures 0.93–0.96; at higher pressures, nitrogen was adsorbed in mesopores and on the external surface of PAF-11 grains (Figure 6a). This finding is also applicable to hybrid membranes, the isotherms of which are plotted in Figure 6b. For the porous framework PAF-11, the apparent surface area SBET = 542 m^2^/g (Equation (3)) and micropore volume (Equation (5)) W = 0.194 cm^3^/g were calculated. These values can be compared with those obtained by Yuan et al. [56] for PAF-11: SBET = 704 m^2^/g and W = 0.203 cm^3^/g (note that the micropore volume in [56] was obtained using the Dubinin–Radushkevich equation).

The obtained isotherms clearly show a decrease in the amount of N_2_ adsorbed on the polymer samples under investigation after the CO_2_-exposure test. In terms of the BET model, this means a decrease in the number of available adsorption sites. In addition, after CO_2_ exposure the initial slope of the isotherms was reduced relative to as-cast films. In other words, Henry’s law constant decreased as a result of conditioning polymer samples with CO_2_.

The calculated adsorption parameters are listed in Table 3. First of all, the apparent BET surface area (i.e., the number of available adsorption sites) of aromatic filler PAF-11 was less than *S*_BET_ for both the neat PTMSP sample and for modified PTMSP ones. The relatively high value of the Henry constant (*K*_H_) for PAF-11 indicates a significant adsorbate–adsorbent interaction. However, for M0, M20, and M30 membranes the Henry constant was approximately constant. This is an interesting fact that could mean “shielding” of PAF-11 adsorption sites (the benzyl rings) by the polymer chains. It can be seen from Table 2, that CO_2_ exposure caused both the BET surface area and the Henry constant to be decreased.

The free-volume fraction of prepared films was approximately the same (≈23%). This value is consistent with the free-volume fraction range (0.20–0.28) obtained using gas and vapor sorption methods [54]. After exposure in CO_2_, the free-volume fraction f of the polymer samples decreased (last column in Table 3). Free-volume reduction (i.e., *f* of the free-volume fractions in unaged and aged samples) was 5.7 (22.8 − 17.1 = 5.7), 4.7, 2.7, and 2.4% for membranes PTMSP, M0, M20, and M30, respectively. It is reasonable to assume that the aging rate is proportional to the *f*. Thus, the neat PTMSP sample should be the most prone to physical aging, M0 should be slightly less receptive, M20 should be much less receptive, and the most unreceptive sample, with the highest content of PAF-11, should be M30. Significantly, the aging of TFC membranes occurred in the same sequence during the long-term gas permeance tests discussed above in Section 3.2.

During constant-pressure CO_2_ exposure, two interrelated processes can occur: swelling of the polymer (at elevated pressures—plasticization) and physical aging (relaxation, shrinkage). The interaction of the adsorbed gas (CO_2_) with the polymer initiates conformational changes in macromolecules, facilitating their rearrangement into a more optimal (equilibrium) supramolecular structure. The balance between the two noted tendencies (swelling and shrinkage) depends, among other things, on the membrane thickness [58]. Conformational rearrangements of macromolecules are more probable for thin polymer films, in which the relative fraction of mobile near-surface macromolecules increases. It is their mobility that is usually associated with the accelerated physical aging of thin polymer films [59,60]. The authors of [58], when studying the permeability of CO_2_ and He through thin PTMSP films, noted that the preliminary exposure of the films to CO_2_ (less than 2 h, at an excess pressure of <1 bar) affected the permeability of helium. It was found that after this procedure, the helium permeability became approximately three times lower compared to the initial permeability. Since the migration of gas molecules through the polymer is carried out along its microvoids (free-volume elements), the decrease in helium permeability indicates a reduction in free volume after exposure to CO_2_. A noticeable decrease in the fraction of free volume is equivalent to accelerated physical aging of the glassy polymer. It can be assumed that such an interpretation is also applicable to our experiments.

## 4. Conclusions

PTMSP dense membranes and thin-film composite membranes (selective layer thickness is about 1 μm) were modified with branched polyethyleneimine—PEI and porous aromatic framework PAF-11 polymer additives. Based on the N_2_ adsorption isotherm data, the percentage of reduction in the free volume of PTMSP was 1.5–3 times higher compared to the modified PTMSP. It was found that in 450 days the permeability of CO_2_ for PTMSP membrane decreased to 14% of the initial value. Modified TFC membranes exhibit slower aging and correspondingly higher permeance compared to a TFC membrane with neat PTMSP selective layer. Relative CO_2_ permeances of the membranes decreased in the series: PTMSP < M0 < M20 < M30. The introduction of crosslinked branched PEI into the PTMSP matrix leads to a certain stabilization of permeation behavior. Blending of PTMSP/PEI with porous filler PAF-11 further slowed the relaxation of TFC membranes: the relative CO_2_ permeances were 42 and 43% over 450-day aging for membranes M20 and M30, respectively. Thus, blending of PTMSP with PAF-11 increased the relative CO_2_ permeance by 28–29% compared to the neat PTMSP composite membrane. After 450 days of aging the permeability of the PTMSP membrane dropped from 75 to 10 m^3^(STP)/(m^2^·h·bar), while the permeability of the membrane containing 30 wt% PAF-11 and PEI decreased from 110 to 47 m^3^(STP)/(m^2^·h·bar). Experimental data were fitted using the Kohlrausch–Williams–Watts function and the limiting (equilibrium) values of the CO_2_ and N_2_ permeances of the TFC membranes were estimated. The relaxation time τ for both permeating gases decreased in the series PTMSP < M0 < M20 < M30. Indeed, the shorter the relaxation time, the higher the aging rate and the faster the gas permeance should decrease. M20 and M30 membranes showed the same limiting relative CO_2_ permeability (*q_eq_* = 31%), which was noticeably (4.4 times) higher than the corresponding value for the unmodified PTMSP membrane. The limit value of CO_2_ permeance for TFC PTMSP membrane as 5.2 m^3^(STP)/(m^2^·h·bar), while the value of 34 m^3^(STP)/(m^2^·h·bar) was reached for a membrane containing 4 wt% cross-linked PEI and 30 wt% PAF-11. Membranes with such stable and high permeability have great prospects as a non-porous support for coating ultrathin selective layers.

## Figures and Tables

**Table 1:** Initial gas permeance, *Q*(0), through the TFC membranes and fitting parameters of “relaxation” Equation (8).

Membrane	*Q*(0) (m^3^ (STP)/m^2^ h bar)		*τ* (days)		*β*		*q_eq_*	
	N_2_	CO_2_	N_2_	CO_2_	N_2_	CO_2_	N_2_	CO_2_
PTMSP	19.4	75.1	53.7	83.3	0.56	0.55	0.04	0.07
M0	13.7	56.9	82.4	145.4	0.53	0.49	0.07	0.15
M20	30.1	94.4	100.9	136.4	0.54	0.53	0.17	0.31
M30	39.0	110.5	103.4	150.9	0.52	0.51	0.13	0.31

M0, M20, and M30 denote the TFC membranes with the selective layers PTMSP/PEI, PTMSP/PEI/20% PAF, and PTMSP/PEI/30% PAF, respectively.

**Table 2:** Comparison of the TFC membranes’ aging.

Selective Layer	Selective Layer Thickness, μm	CO_2_ Permeance of Fresh As-Cast Membrane, GPU	Aging Time (Ambient Conditions)	CO_2_ Permeance *Q*/*Q*_0_, %	Reference
PTMSP	1.7	1700	>600 days	8.5	[35]
	6.8	6500		5.5	
PIM-1/C-HCP	2.0	11,500	100 days	81	[41]
PIM-1	0.3	8000	90 days	3.7	[42]
PIM-1	0.7	4300	56 days	11	[39]
PIM-1/MOF-74-Ni		5000		24	
PIM-1/NH2-UiO-66		7500		12	
PTMSP + PEI	1.2	15,100	>425 days	23	[39]
PTMSP + PEI + 10% IR-PAN-a	1.8	23,700		17	
PTMSP + PEI + 20% IR-PAN-a		24,700		26	
PTMSP + PEI + 30% IR-PAN-a		24,100		22	
PTMSP + PEI + 10% IR-PAN-aM	1.0	20,900		30	
PTMSP + PEI + 20% IR-PAN-aM		24,500		27	
PTMSP + PEI + 30% IR-PAN-aM		25,100		27	
Carbon molecular sieves (PDMS pyrolysis) precursor	0.087	239	45 days	9.6	[43]
Carbon molecular sieves (PDMS pyrolysis) 500 °C	0.069	294		9.9	
Carbon molecular sieves (PDMS pyrolysis) 600 °C	0.082	320		0.9	
Carbon molecular sieves (PDMS pyrolysis) 700 °C	0.072	8		17.5	
PU/PIM-1	30	11	60 days	82	[44]
PTMSP	1.0	27,700	450 days	14	this work
PRMSP + PEI	1.1	21,000		30	
PTMSP + PEI + 20% PAF-11	1.0	34,400		42	
PTMSP + PEI + 30% PAF-11	1.1	40,600		43	

**Table 3:** The parameters of PTMSP-based films from N_2_ adsorption isotherms: apparent BET surface area (SBET)and the Henry constant (*K*_H_). The skeletal density (*ρ_sk_*) and calculated free-volume fraction (*f*) are also presented. The results for the porous aromatic framework PAF-11 are also presented. The parameters after CO_2_ exposure are shown in the brackets.

Sample	*S*_BET_ (m^2^/g)	*K*_H_ (mol/g)	*ρ_sk_* (g/cm^3^)	*f* (%)
PTMSP	816 (589)	5.3 (2.3)	1.007 (0.997)	22.8 (17.1)
M0	829 (648)	4.2 (3.3)	0.985 (1.011)	22.7 (19.0)
M20	787 (747)	4.3 (4.2)	1.039 (0.934)	22.7 (20.0)
M30	839 (764)	4.0 (4.2)	1.005 (0.956)	23.2 (20.8)
PAF-11	542 (-)	12.0 (-)	1.185 (-)	18.7 (-)

## Data Availability

Not applicable.
